# Primary Prevention Implantable Cardiac Defibrillators: A Townsville District Perspective

**DOI:** 10.3389/fcvm.2020.577248

**Published:** 2020-10-27

**Authors:** Nathan Engstrom, Geoffrey P. Dobson, Kevin Ng, Hayley L. Letson

**Affiliations:** ^1^College of Medicine & Dentistry, Heart, Trauma and Sepsis Research Laboratory, James Cook University, Townsville, QLD, Australia; ^2^Cardiac Investigations, The Townsville University Hospital, Douglas, QLD, Australia; ^3^Cardiology Clinic, Cairns Hospital, Cairns, QLD, Australia

**Keywords:** heart failure, implantable cardiac defibrillator, primary prevention, arrhythmia, sudden cardiac death, shocks

## Abstract

**Background:** Despite major advances in treating patients with severe heart failure, deciding who should receive an implantable cardiac defibrillator (ICD) remains challenging.

**Objective:** To study the risk factors and mortality in patients after receiving an ICD (January 2008–December 2015) in a regional hospital in Australia.

**Methods:** Eighty-two primary prevention patients received an ICD for ischemic cardiomyopathy (ICM, *n* = 41) and non-ischemic cardiomyopathy (NICM, *n* = 40) with 4.8-yrs follow-up. One patient had mixed ICM/NICM indications. Ventricular arrhythmias were assessed using intracardiac electrograms. Statistical analysis compared the total population and ICM and NICM groups using Kaplan-Meier for survival, Cox regression for mortality predictors, and binary logistic regression for predictors of ventricular arrhythmias (*p* < 0.05).

**Results:** Major risk factors were hypercholesterolemia (70.7%), hypertension (47.6%), and obesity (41.5%). Severe obstructive sleep apnea (OSA) was found exclusively in NICM patients (23.7%, *p* = 0.001). Mortality was 30.5% after 4.8-yrs. The majority of patients (n=67) had no sustained ventricular arrhythmias yet 28% received therapy (*n* = 23), 18.51% were appropriate (*n* = 15), and 13.9% inappropriate (*n* = 11). Patients receiving ≥2 incidences of inappropriate shocks were 18-times more likely to die (*p* = 0.013). Three sudden cardiac deaths (SCD) (3.7%) were prevented by the ICD.

**Conclusion:** Patients implanted with an ICD in Townsville had 30.5% all-cause mortality after 4.8-yrs. Only 28% of patients received ICD therapy and 13.9% were inappropriate. OSA may have contributed to the fourfold increase in inappropriate therapy in NICM patients. Our study raises important efficacy, ethical and healthcare cost questions about who should receive an ICD, and possible regional and urban center disparities.

## Introduction

Heart failure (HF) represents a global healthcare pandemic that affects over 26 million people worldwide including 5.8 million in the United States, 10 million in Europe, up to 9 million in South East Asia, and around 300,000 in Australia (1–4). Despite major advances in patient management and drug therapies, treating patients with severe HF remains challenging. The problem is formidable given that 30–50% of this group will experience a cardiac arrest (5), which has an out-of-hospital survival rate of <10% (3, 5). To address this problem, 40 years ago Mirowski and colleagues were among the first to introduce implantable cardiac defibrillators (ICDs) into cardiac arrest patients as a preventive measure to reduce further attacks (6), which has now become a global standard practice in developed countries (7, 8).

Today, ICDs are also recommended for primary prevention patients with ischemic cardiomyopathy (ICM), usually from a previous myocardial infarction, and patients with non-ischemic cardiomyopathy (NICM) of a dilated or non-dilated origin with a left ventricular ejection fraction (LVEF) ≤35% (3, 5, 9, 10). ICM is the most common type of dilated cardiomyopathy where the left ventricle has been enlarged, dilated, and weakened usually from ischemia associated with coronary artery disease and myocardial infarction. NICM is defined as disease of the myocardium associated with mechanical or electrical dysfunction from ventricular hypertrophy or dilatation with a strong genetic component (3, 10, 11).

Notwithstanding the small clinical risks of device insertion (12), the most common problems of the ICD include lead failure, premature battery life, failure of algorithms to discriminate ventricular from supraventricular arrhythmias, device activation when shocks are not required, and patient anxiety issues (e.g., anticipatory “phantom shocks”) (8, 13). Despite the potential of ICDs to save lives, there is increasing controversy about who should receive a device, and their clinical efficacy (8, 14). Differences in patient outcomes appear to reflect the complexity of multiple phenotypes of heart failure with different comorbidities, the selection criteria based on ejection fraction classification, and lack of consensus guidelines for patient selection (4, 8, 13, 15). The aim of this study was to define risk factors, arrhythmia incidence, and mortality in primary prevention heart failure patients in the Townsville district in northern Australia. Secondary objectives were to investigate differences between ICM and NICM patients, and to compare findings with published data from larger metropolitan centers. This is, to the best of our knowledge, the first such analysis of ICD patients in regional Australia.

## Materials and Methods

### Ethical Considerations

This study was conducted in full conformance with the principles of the National Statement on Ethical Conduct in Human Research (Updated 2018). The study was approved by the Townsville Hospital and Health Service (THHS) Human Research Ethics Committee (HREC) (HREC/18/QTHS/57) and James Cook University HREC (H7438).

### Study Population

The study population consisted of 82 patients within the THHS District who had a primary prevention ICD implanted at Townsville University Hospital (TUH) between January 1^st^, 2008 and December 31^st^, 2015. The study population included new implants and generator changes, which in a few cases required a device history prior to 2008. Primary prevention ICD generator changes were included as this reflects normal clinical practice.

Primary prevention patients who had an ICM or NICM indication were included for analysis, while all secondary prevention ICD patients were excluded. Angiography was used to exclude coronary artery disease as a cause of cardiomyopathy in NICM patients. All patients had coronary angiography with the exception of five patients who had a genetic or familial indication for ICD, e.g., long QT syndrome, non-compaction, sarcoidosis. In order to reduce the heterogeneity of non-ischemic group we identified and separated channelopathy patients with preserved ejection fraction. Exclusion criteria included incomplete chart information or ICD follow-up data. Patients were identified as indicated by implant report or clinic letter by the implanting Cardiac Electrophysiologist. Primary prevention indications were those with a history of coronary artery disease (CAD) or myocardial infarction, or the absence of CAD with an LVEF ≤35% on maximal medical therapy and New York Heart Association (NYHA) functional class II-III, or NYHA class 1 and an LVEF ≤30%. There was no prior history of sustained ventricular arrhythmias subsequent to ICD implantation in this patient cohort.

### Data Collection and Analyses

Data were retrospectively collected from TUH's medical records and Cardiobase (version 8.1) from which clinic letters and ICD follow-up information was obtained (Public Health Act Approval: RD007511). Demographic information, comorbidities, ejection fraction (EF), and medications prior to ICD implantation were extracted and recorded. Obesity status was indicated by clinic letter or with a body mass index (BMI) ≥30. New implant device follow-up information, including complications and all-cause mortality, was collected from time of implant up until December 31^st^, 2018. For patients who received an ICD generator change, follow-up details were included from the previous devices to obtain a complete ICD history.

### Device Settings

All devices were programmed at the discretion of the implanting cardiac electrophysiologist and had atrial arrhythmia algorithms turned on. Subcutaneous ICDs had a shock zone from 220–240 bpm and a conditional shock zone from 180–200 bpm with full output shocks programmed. Devices from Boston Scientific, Abbott, and Biotronik predominantly had a two-zone active therapy configuration with a monitor zone, whereas Medtronic devices mostly had three active therapy zones and a monitor zone programmed. Examples of short, intermediate, and long duration detection endocardial device settings can be found in Table 1.

**Table 1:** Endocardial device detection settings.

**Duration**	**VF zone**	**FVT zone**	Third active zone^*^	
			**VT zone**	**Monitor zone**
Short	194–240 bpm	170–222 bpm	150–182 bpm	110–171 bpm
	12–18 intervals/1 s detection	12–24 intervals/1–4 s detection	12–20 intervals/2.5–15 s detection	20–32 intervals/2.5–15 s detection
	ATP while charging	2–10 ATP	6–8 ATP	No therapy
	Max shocks	Max shocks	Max shocks	
Intermediate	220–250 bpm	170–222 bpm	140–188 bpm	133–170 bpm
	12–24 intervals/2.5–5 s detection	20–32 intervals/2.5–5 s detection	16–24 intervals/7–10 s detection	28–32 intervals/11–60 s detection
	ATP while charging	1–10 ATP	2–8 ATP	No therapy
	Max shocks	Max shocks	Max shocks	
Long	222–250 bpm	171–214 bpm	140–185 bpm	133–170 bpm
	20–30 intervals/2.5–10 s detection	30–48 intervals/7–10 s detection	18–30 intervals/6–10 s detection	32–48 intervals/11–60 s detection
	ATP while charging	2–8 ATP	2–8 ATP	No therapy
	Max shocks	Max shocks	Max shocks	

* *If programmed. VF, ventricular fibrillation; FVT, fast ventricular tachycardia; VT, ventricular tachycardia; ATP, antitachycardia pacing therapy*.

### Interpretation of ICD Therapy

Therapy was deemed “appropriate” for ventricular arrhythmias or “inappropriate” for supraventricular arrhythmias or device and/or lead malfunction. The terms “therapy” in this study refer to anti-tachycardia pacing (ATP) and shocks, and “shocks” refer to defibrillation from the ICD. Interpretation of therapy occurred in one of two ways: (1) From electrocardiograms in the clinical letters, which were interpreted by a certified cardiac device specialist (CCDS) and checked by a consultant medical specialist (KN), or (2) from individual device intracardiac electrograms of patients that were not found in the clinical letters, and interpreted by the same investigators (CCDS and KN). Single chamber devices were interpreted using onset and stability as well as electrogram morphology match score. When this was not optioned on the device the high voltage electrogram (far field) was analyzed for changes during onset and offset to identify changes to distinguish supraventricular tachycardia from ventricular tachycardia. For dual chamber devices ventricular tachycardia was identified by ventricular electrogram rate greater than atrial electrogram rate (V>A). When tachycardia was 1:1, the chamber of tachycardia onset was used together with onset (gradual or sudden) and stability (regular or irregular). Electrogram morphology was also used as a discriminator. As for single chamber devices, if unavailable the high voltage electrogram was assessed to differentiate supraventricular from ventricular tachycardia.

### Sudden Cardiac Death Criteria

Sudden cardiac death was defined as spontaneously occurring ventricular tachycardia or ventricular fibrillation >240 beats per minute based on published work (16). If ventricular tachycardia was caused by anti-tachycardia pacing leading to an acceleration of heart rate >240 it was excluded from our criteria of sudden cardiac death. ICD intracardiac electrograms were assessed to identify patients who met this criterion in our study. This criterion relates to arrhythmic death only and does not account for all cases of sudden death that may also have occurred from non-arrhythmogenic pump failure because of the severely reduced LVEF found in this patient population (16).

### Statistical Analysis

Statistical analysis was conducted using IBM SPSS version 24. Descriptive analysis is reported as frequencies, mean (standard deviation) or median (interquartile range). Variables were compared within the total population, and between ICM and NICM patients with Chi-squared test for categorical data and analysis of variance (ANOVA) with Levene's test of homogeneity of variance for continuous variables. One patient had a mixed indication for ICD and was not included in ICM vs NICM analysis. Kaplan–Meier test was used for survival analysis, with log-rank test for comparison between ICM and NICM patients. Cox proportional hazard analysis was performed to determine predictors of mortality, and binary logistic regression was used to assess associations between patient variables and appropriate ICD therapy. Multivariate regression analysis was conducted for parameters showing statistical significance in univariate tests. A *p* < 0.05 was considered statistically significant.

## Results

### Clinical Characteristics

The mean age of patients was 59 ± 16 years although the NICM patient cohort was significantly younger (55 ± 18 years, p=0.045). A significantly higher proportion of males received ICDs in this district (81.7%; p=0.011). There was an even distribution of ICM and NICM patients (50 vs. 48.8%, *p* = 0.912; Table 2). Only three patients were identified as Indigenous. Risk factors in our cohort included hypercholesterolemia (70.7%, *p* < 0.05), hypertension (47.6%, *p* < 0.05), obesity (41.5%), and Type 2 diabetes (29.3%) (Table 2). ICM patients had higher hypercholesterolemia (90%; *p* = 0.002), higher smoking history, coronary artery bypass grafting, valve surgery, and percutaneous coronary intervention (Table 2). Severe obstructive sleep apnea was found exclusively in the NICM group (23.7%, *p* = 0.001; Table 2). Most patients received beta blockade medical therapy (90.2%) with more ICM patients receiving statins (95.1%, *p* < 0.001) and antiplatelet (78%, *p* = 0.003) medications (Table 3). Diuretics and ACE inhibitors were commonly prescribed, with 34.1% of all patients receiving an anticoagulant. Nitroglycerine was more commonly prescribed in patients with coronary artery disease (19.5%, *p* = 0.030) compared to those with a NICM (Table 3).

**Table 2:** Demographics and risk factors of primary prevention heart failure patients.

**Parameter**	**Total population**	**ICM**	**NICM**	***P*-value**
Age (yrs)	59 ± 16	62 ± 12	55 ± 18	0.045
**Gender**				
Female	15 (18.3%)	3 (7.3%)	12 (3%)	0.011
Male	67 (81.7%)^†^	38 (92.7%)	28 (70%)	
Indigenous	3 (3.7%)^†^	2 (4.9%)	1 (2.5%)	–
Height (cm)	173 ± 9	175 ± 7	171 ± 10	0.129
Weight (kg)	89 ± 22	89 ± 19	90 ± 25	0.936
BMI	29 ± 6	29 ± 5	30 ± 7	0.674
LVEF (%)	29 ± 13	27 ± 8	31 ± 17	0.759
Cardiomyopathy type	ICM: 41 (50%)	41 (50%)	40 (48.8%)	0.912
	NICM: 40 (48.8%)		Dilated cardiomyopathy: 31 (37.8%)	
			Mixed: 1 (1.2%)	
			Channelopathy: 2 (2.4%)	
			HCM: 4 (4.9%)	
			LV non compaction: 1 (1.2%)	
			Sarcoidosis: 1 (1.2%)	
Hypertension	39 (47.6%)	23 (57.5%)	16 (42.1%)	0.275
Type 2 diabetes	24 (29.3%)^†^	13 (32.5%)	11 (29%)	0.809
Hypercholesterolemia	58 (70.7%)^†^	36 (90%)	22 (58%)	0.002
OSA	9 (11%)	0 (0%)	9 (23.7%)	0.001
Obesity	34 (41.5%)	19 (46.3%)	15 (38.5%)	0.506
Alcohol abuse	14 (17.1%)	4 (10%)	10 (26.3%)	0.079
COPD	15 (18.3%)	11 (27.5%)	4 (10.5%)	0.084
**Smoking status**				
Current	9 (11 %)	6 (15%)	3 (7.9%)	–
Former	25 (30.5%)	18 (45%)	7 (18.4%)	
Unknown	5 (6.1%)	3 (7.5%)	2 (5.2%)	
**Atrial fibrillation**				
Chronic	9 (11%)	3 (7.3%)	6 (15.8%)	–
PAF	26 (31.7%)	16 (39%)	10 (26.3%)	
**CABG**				
Primary	23 (28%)	23 (57.5%)	0 (0%)	–
Redo	1 (1.2%)	1 (2.5%)	0 (0%)	
**PCI with stent**				
Single	5 (6.1%)	4 (40%)	1 (2.6%)	–
x2 or more	8 (9.8%)	8 (20%)	0 (0%)	
**Valve replacement**				
Mitral	2 (2.4%)	2 (5%)	0 (0%)	–
Aortic	3 (3.7%)	2 (5%)	1 (2.5%)	

Data presented as total (%) or mean ± standard deviation.

† *p < 0.05. The patient with mixed indication was Male, Non-Indigenous, 166 cm, 77 kg, with a BMI of 31 and LVEF of 25%, and the following comorbidities: Hypertension, Hypercholesterolemia, Obesity, COPD, Former smoker and PCI with single stent. ICM, Ischemic Cardiomyopathy; NICM, Non-Ischemic Cardiomyopathy; BMI, Body Mass Index; LVEF, Left Ventricular Ejection Fraction; LV, Left Ventricle; HCM, Hypertrophic Cardiomyopathy; OSA, Obstructive Sleep Apnea; COPD, Chronic Obstructive Pulmonary Disease; CABG, Coronary Artery Bypass Graft; AF, Atrial Fibrillation; PAF, Paroxysmal Atrial Fibrillation*.

**Table 3:** Medications for the study cohort.

**Medication**	**Total population**	**ICM**	**NICM**	**P-value**
Diuretics	57 (69.5%)^†^	33 (80.5%)	24 (63.2%)	0.131
ACE	57 (69.5%)^†^	31 (75.6%)	26 (70.2%)	0.619
Angiotensin 2 agonist	12 (14.6%)	8 (19.5%)	4 (10.5%)	0.353
B-blockers	74 (90.2%)^†^	39 (95.1%)	35 (92.1%)	0.667
Ca-blockers	7 (8.5%)	5 (12.2%)	2 (5.3%)	0.434
Statins	60 (73.2%)^†^	39 (95.1%)	21 (55.3%)	<0.001
Insulin	10 (12.2%)	5 (12.2%)	5 (13.2%)	1.000
Non-insulin diabetic (Metformin/Diamicron)	15 (19.0%)	7 (17%)	8 (21%)	0.776
GTN	9 (11.0%)	8 (19.5%)	1 (2.63%)	0.030
Digoxin	20 (24.4%)	12 (29.3%)	8 (21.1%)	0.447
Antiplatelet	49 (59.8%)^†^	32 (78%)	17 (44.7%)	0.003
Anticoagulant	28 (34.1%)	15 (36.6%)	13 (34.2%)	1.000
Amiodarone	9 (11%)	5 (12.2%)	4 (10.5%)	1.000

*Data presented as total (%). Medications for the patient with mixed indication included a diuretic, ACE, statin, digoxin and antiplatelet. P-value represents between-groups difference*.

† *p < 0.05. GTN, Nitroglycerine; ACE, Angiotensin Converting Enzyme*.

The mean follow-up time following ICD implantation was 4.8 ± 3 years (range: 0–13 years). New implants were received by 76.8% of patients and 20.7% had a generator change (Table 4). ICDs included single chamber devices (40.2%) and dual (35.4%). Cardiac resynchronisation therapy with defibrillation (CRT-D) was fourfold higher in the NICM than ICM patients (12 vs. 3) (Table 4).

**Table 4:** Device type and implant type for the primary prevention patients.

**Parameter**	**Total population**	**ICM**	**NICM**	***P*-value**
Length of follow-up (years)	4.8 ± 3 (0–13)	4.8 ± 3 (1–13)	4.9 ± 3 (1–12)	0.954
**Device type**				
Single chamber	33 (40.2%)	20 (49%)	12 (30%)	–
Dual chamber	29 (35.4%)	16 (39%)	13 (32.5%)	
PPM upgrade to dual chamber ICD	1 (1.2%)	1 (2.4%)	0 (0%)	
PPM Upgrade to CRT-D	1 (1.2%)	0 (0%)	1 (2.4%)	
Sub-Q ICD	3 (3.7%)	1 (2.4%)	2 (5%)	
CRT-D	15 (18.3%)	3 (7.3%)	12 (30%)	
**Implant type**				
New implant	63 (76.8%)^†^	33 (80.5%)	30 (75%)	–
Generator change	17 (20.7%)	7 (17.1%)	9 (22.5%)	
Generator change with new HV Lead	2 (2.4%)	1 (2.4%)	1 (2.5%)	
Upgrade to CRT-D	3 (3.7%)	0 (0%)	3 (7.5%)	
Downgrade to PPM	1 (1.2%)	1 (2.4%)	0 (0%)	
CTS LV lead placement	1 (1.2%)	0 (0%)	1 (2.5%)	

Data presented as total (%) or mean ± standard deviation (range) for length of follow-up. P-value represents between-groups difference.

† *p < 0.05. The patient with mixed indication had CRT-D with CTS LV lead placement. ICM, Ischemic Cardiomyopathy; NICM, Non-Ischemic Cardiomyopathy; PPM, Permanent Pacemaker; ICD, Implantable Cardiac Defibrillator; CRT-D, Cardiac Resynchronisation Therapy with Defibrillator; Sub-Q ICD, Subcutaneous Implantable Cardiac Defibrillator; PCI, Percutaneous Coronary Intervention; CTS, Coronary Thoracic Surgery; LV, Left ventricle*.

**Figure 1:**
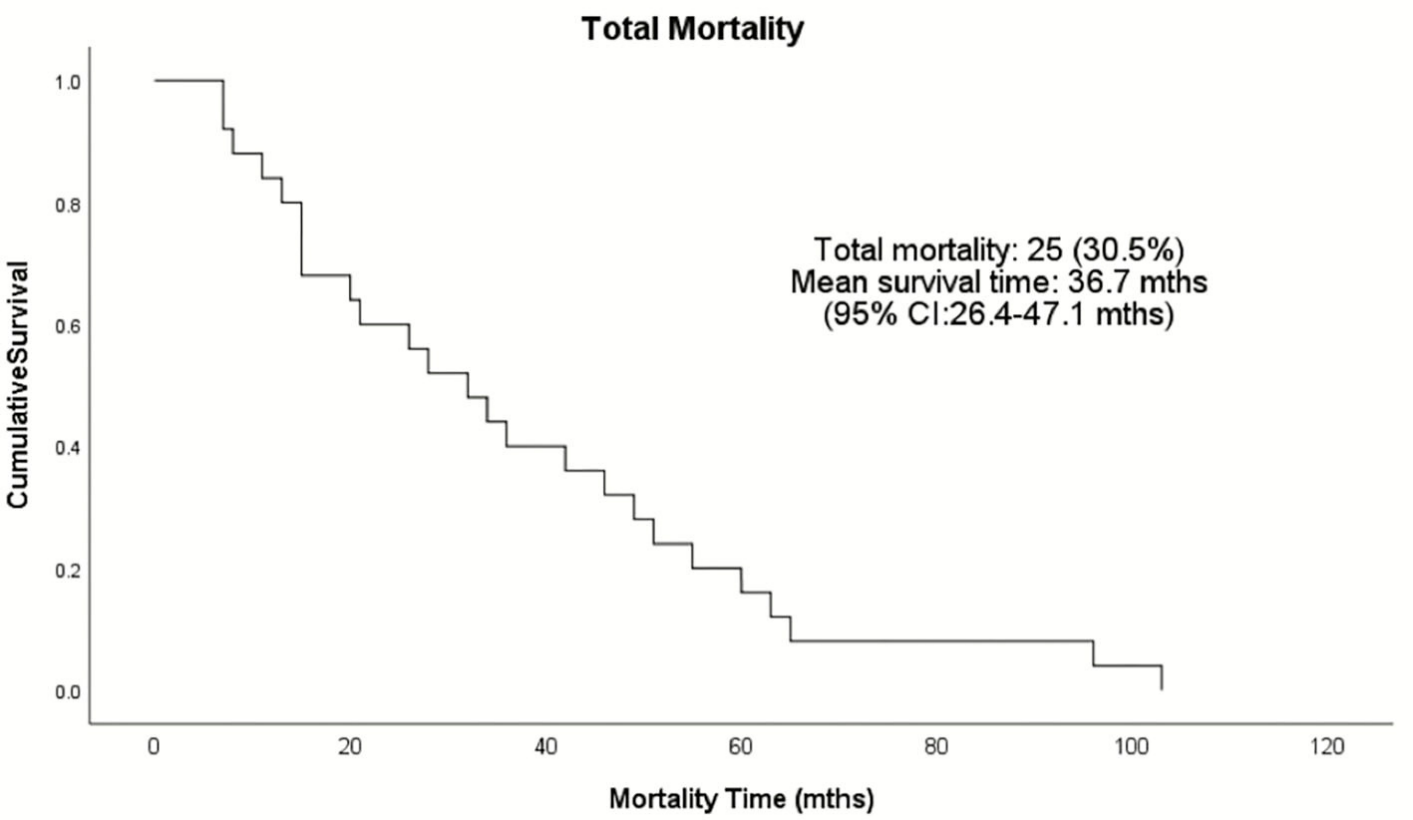
Kaplan–Meier survival analysis of the total patient population receiving ICDs in the Townsville District from January 2008–December 2015. Twenty-five patients died during the follow-up period as represented by the events on the Kaplan-Meier cumulative survival curve.

**Figure 2:**
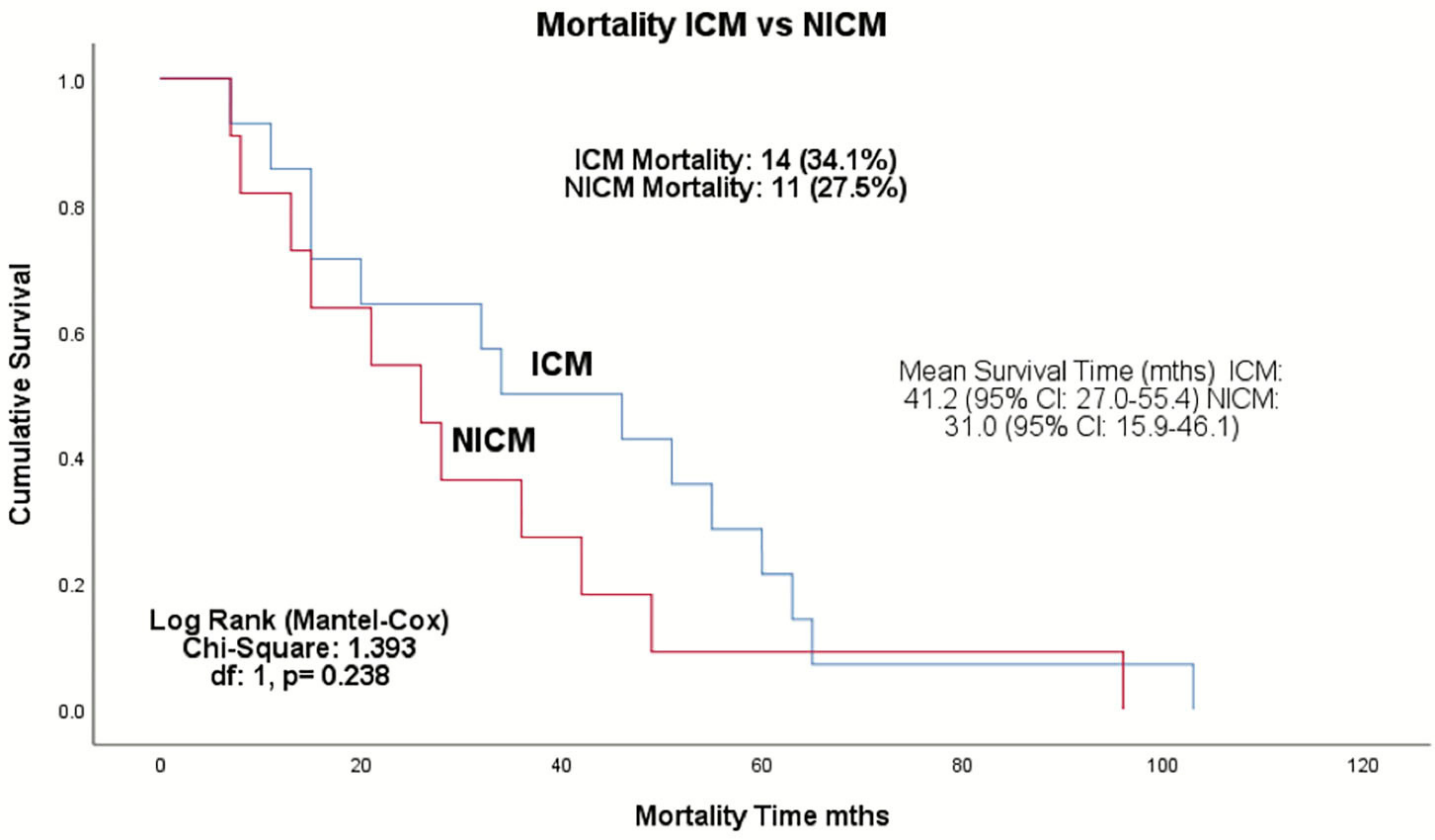
Kaplan–Meier survival analysis of the ICM and NICM patient cohorts in the Townsville District over 4.8-years follow-up from January 2008–December 2015. Log rank test shows no significant difference in survival between ICM and NICM patients (*p* = 0.238). ICM, ischemic cardiomyopathy; NICM, non-ischemic cardiomyopathy.

Complications occurred in 13% of total patients (8 were ICM and 3 NICM patients, *p* = 0.116; Table 5). Two patients had lead revision, failed LV placement, and lead failure post-implant, and one patient had an infection, lead perforation, wound flush-and-clean and pre-implant arrest during the anesthetic induction. Twenty-five patients (30.5%) died during the follow-up period, with a mean survival time of 36.7 months (range: 26.4–47.1 months) (Figure 1). The ICM group had a 10-month longer survival time compared to the NICM group (41.2 vs. 31.0 months) but this was not significant [Log Rank (Mantel Cox) χ^2^ = 1.393, df = 1, *p* = 0.238; Figure 2].

**Table 5:** Mortality, procedural complications, and therapy following implant.

**Parameter**	**Total population**	**ICM**	**NICM**	**P-value**
Time to mortality (months)^†^	32 (7–103)	40 (7–103)	23 (7–96)	0.267
Total complications	11 (13.3%)	3 (7.3%)	8 (20%)	0.116
**Complications**				
•Lead revision	2 (2.4%)	0 (0%)	2 (5%)	–
•Infection	1 (1.2%)	0 (0%)	1 (2.5%)	
•Failed LV placement	2 (2.4%)	1 (2.4%)	1 (2.5%)	
•Lead perforation	1 (1.2%)	0 (0%)	1 (2.5%)	
•Wound flush and clean	1 (1.2%)	0 (0%)	1 (2.5%)	
•Lead failure post-implant	2 (2.4%)	1 (2.4%)	1 (2.5%)	
•Pre-implant arrest during anesthetic induction	1 (1.2%)	1 (2.4%)	0 (0%)	
Total therapy (ATP and shocks)	23 (28.0%)	8 (19.5%)	15 (37.5%) 12 DCM (30%) 2 HCM (5%) 1 Channelopathy (2.5%)	0.088
Appropriate therapy (ATP and Shocks)	15 (18.5%)	6 (14.6%)	9 (22.5%)	0.387
	12 (Single chamber)		6 DCM (15%)	
	3 (Dual chamber)		1 HCM (2.5%)	
			1 Sarcoidosis (2.5%)	
			1 Channelopathy (2.5%)	
Inappropriate therapy (ATP and Shocks)				<0.001
•AF/SVT	10 (12.6%)	3 (7.3%)	7 (18.4%)	
	4 (Single chamber)	0 (0%)	5 DCM (12.5%)	
	6 (Dual chamber)		2 HCM (5%)	
•Lead malfunction	1 (1.3%)		1 (2.6%)	
	1 (Dual chamber)		1 DCM (2.6%)	
Number of shocks in lifetime^†^	0 (0–55)	0 (0–55)	0 (0–18)	0.089
Appropriate shock therapy	6 (6.1%)	3 (7.3%)	3 (7.5%) 2 DCM (5%) 1 Channelopathy (2.5%)	0.114
Inappropriate shock therapy	10 (12.3%)	2 (4.9%)	8 (20%) 6 DCM (15%) 2 HCM (5%)	0.039
**Shock therapy**				
•Appropriate VT	3 (3.7%)	1 (2.4%)	2 (5.3%)	–
			2 DCM (5.3%)	
•Appropriate VF	2 (2.4%)	1 (2.4%)	1 (2.6%)	
			1 Channelopathy (2.6%)	
	1 (1.2%)	1 (2.4%)	0 (0%)	
•VT storm	8 (9.8%)	2 (4.9%)	6 (15.8%)	
•Inappropriate x1			4 DCM (10%)	
			2 HCM (5%)	
	2 (2.4%)	0 (0%)	2 (5.3%)	
•Inappropriate x2 or more			2 DCM (5.3%)	
Number of ATP in lifetime^†^	0 (0–167)	0 (0–167)	0 (0–13)	0.305
Anti-tachycardia pacing				0.001
•Appropriate	13 (15.8%)	5 (12.1%)	8 (21%)	
			6 DCM (15%)	
			1 HCM (2.5%)	
			1 Sarcoidosis (2.5%)	
	2 (2.4%)	1 (2.4%)	1 (2.6%)	
Inappropriate			1 DCM (2.6%)	
Sudden cardiac death criteria (VT/VF >240 bpm)	3 (3.7%)	2 (4.9%)	1 (2.5%) 1 Channelopathy (2.5%)	0.571

*Data presented as total (%) or median (range)^†^. ICM, Ischemic Cardiomyopathy; NICM, Non-Ischemic Cardiomyopathy; BMI, Body Mass Index; LVEF, Left Ventricular Ejection Fraction; LV, Left Ventricle; HCM, Hypertrophic Cardiomyopathy; DCM, Dilated Cardiomyopathy; VT, Ventricular Tachycardia; VF, Ventricular Fibrillation; AF, Atrial Fibrillation; SVT, Supraventricular Tachycardia; ATP, Anti-tachycardia Pacing; Single chamber device; Dual chamber device*.

### Risk Factors for Mortality and ICD Events

Age, gender, BMI, and ejection fraction were not significant predictors of mortality in this patient population (Table 6). Smokers were at a significantly higher risk of dying than non-smokers (HR: 4.830, 95% CI: 1.229–18.985, *p* = 0.024; Table 6). Aortic valve replacement carried a ~12-fold increased risk of death (*p* = 0.052) and mitral valve replacement a 4-fold risk (*p* = 0.059; Table 6). Patients receiving two or more incidences of inappropriate shocks were 18-fold more likely to die (HR: 18.286, 95% CI: 1.833–182.387, *p* = 0.013; Table 6). Interestingly, patients who experienced complications had a 73% reduced risk of mortality (HR: 0.275, 95% CI: 0.092–0.821, *p* = 0.021). Patients with obstructive sleep apnea had a 70% reduced risk (Table 6). Multivariate analysis of smoking history, inappropriate shocks, and valve replacement surgery showed a significant increase in mortality risk (χ^2^ = 17.601, df = 8, *p* = 0.024; Table 6).

**Table 6:** Cox proportional hazard model for predictors of mortality.

**Variable**	**Hazard ratio (95% CI)**	***P*-value**
Female gender	0.918 (0.236–3.129)	0.891
Age	0.993 (0.948–1.039)	0.748
BMI	1.028 (0.971–1.088)	0.342
LVEF%	1.007 (0.955–1.061)	0.799
ATP in life-time	0.993 (0.980–1.007)	0.318
Complications	0.275 (0.092–0.821)	0.021
Mitral valve replacement^†^	4.374 (0.985–19.426)	0.052
Aortic valve replacement^†^	11.563 (0.911–146.720)	0.059
CABG	0.387 (0.046–3.224)	0.380
Hypertension	1.489 (0.583–3.806)	0.406
Diabetes	1.327 (0.583–3.021)	0.500
Hypercholesterolemia	1.199 (0.352–4.092)	0.772
OSA	0.301 (0.080–1.141)	0.077
Obesity	0.626 (0.265–1.483)	0.287
Alcohol abuse	0.804 (0.262–2.468)	0.703
COPD	1.250 (0.458–3.413)	0.663
Ever smoked	0.842 (0.363–1.954)	0.689
Current smoker^†^	4.830 (1.229–18.985)	0.024
Any AF	0.806 (0.351–1.851)	0.611
Chronic AF	0.863 (0.356–2.092)	0.774
Paroxysmal AF	1.333 (0.414–4.290)	0.630
Inappropriate therapy (Shock)	1.007 (0.334–3.036)	0.990
Inappropriate therapy (x2 or >)^†^	18.286 (1.833–182.387)	0.013

*CI, confidence interval; BMI, body mass index; LVEF, left ventricular ejection fraction; ATP, Anti-tachycardia pacing; CABG, cardiopulmonary bypass graft; OSA, obstructive sleep apnea; COPD, chronic obstructive pulmonary disease; AF, atrial fibrillation. Results represent univariate analysis. Multivariate analysis was conducted using variables “Current Smoker” and “Inappropriate Therapy (x2 or >)” which showed significantly increased hazard ratio for mortality, as well as “Mitral Valve Replacement” and “Aortic Valve Replacement,” which approached statistical significance (p = 0.052 and p = 0.059, respectively)*.

† *Multivariate analysis: χ^2^ = 17.601, df = 8, p = 0.024*.

Twenty-eight percent (*n* = 23) of all patients received therapy, either pacing or defibrillation (Table 5). Almost 40% of NICM patients received therapy compared to ~20% of ICM patients (*p* = 0.088). Of all patients who received therapy only 18.51% were appropriate for sustained ventricular arrhythmias. While 22.5% of NICM patients received appropriate therapy, 21.05% also received inappropriate therapy, which was significantly higher than in ICM patients (*p* = <0.001). Sudden cardiac death would have occurred in three patients (3.7%), two ICM and one NICM patient, if ICD defibrillation had not occurred (Table 5).

There were no significant associations between patient demographics or risk factors and ventricular arrhythmias (Table 7). However, if patients had a previous smoking history, they were 2.7 times more likely to receive an appropriate shock compared with patients who have never smoked (OR: 2.719, 95% CI: 0.688–10.75, *p* = 0.154). Patients with hypercholesterolemia also showed a two-fold increased likelihood of receiving therapy for ventricular arrhythmias when compared to patients with normal cholesterol levels (OR: 2.083, 95% CI: 0.592–7.327, *p* = 0.252; Table 7).

**Table 7:** Univariate binary logistic regression for appropriate therapy.

**Variable**	**Odds ratio (95% CI)**	***P*-value**
Female gender	1.425 (0.340–5.970)	0.628
Age	0.995 (0.960–1.032)	0.798
Hypertension	0.831 (0.252–2.743)	0.760
Diabetes	1.000 (0.275–3.634)	1.000
Hypercholesterolemia	2.083 (0.592–7.327)	0.253
OSA	1.684 (0.192–14.752)	0.638
BMI	1.039 (0.947–1.141)	0.418
Obesity	0.838 (0.254–2.763)	0.771
Alcohol abuse	1.245 (0.244–6.367)	0.792
COPD	1.375 (0.271–6.980)	0.701
Ever smoked	2.719 (0.688–10.751)	0.154
AF	0.632 (0.191–2.086)	0.451
LVEF %	1.024 (0.983–1.067)	0.257
Complications	0.825 (0.157–4.346)	0.820

*CI, confidence interval; OSA, obstructive sleep apnea; BMI, body mass index; COPD, chronic obstructive pulmonary disease; AF, atrial fibrillation; LVEF, left ventricular ejection fraction*.

## Discussion

The management of ventricular arrhythmias in heart failure patients remains challenging despite significant advances in ICD innovation, clinical research and cardiovascular-modifying drug therapies (7). We report that ICD patients serviced by the Townsville University Hospital monitored over a 4.8-year period had 30.5% all-cause mortality. We also report that 82% (67 patients) experienced no sustained ventricular arrhythmias, yet 28% (23 patients) received therapy. Eighteen per cent of device therapy were appropriate and 14% were inappropriate. No associations were found between ventricular arrhythmias and ejection fraction, hypercholesterolemia, hypertension or obesity. In NICM patients, sleep apnea was associated with a fourfold increase in inappropriate shocks compared to ICM patients. Interestingly, the overall risk of mortality was 73% lower in patients who had complications, which may be due to increased follow-up and medical care in this subset. Three patients would have almost certainly died without an ICD. These data raise a number of important questions about the utility and effectiveness of ICDs and the current guidelines and selection criteria on who should receive a device.

### Mortality Was Higher in Regional North Queensland

All-cause mortality of 30.5% in the Townsville district was 1.2 to 2 times higher than mortality reported in other cohort studies in Australia, New Zealand, North America and Europe (15, 17–20). Although we found no significant difference in mortality between the ICM and NICM patients, the total mortality in ICM patients was 1.4 to 2.4 times those in the MADIT, MADIT II, and MUST trials (21, 22), and 1.3 to 2.8 times higher in the NICM patients compared to the CAT, AMIOVIRT, DEFINITE, and recent DANISH trials (23–26).

Currently, we do not know the reasons for the higher mortality but it may relate to differences in patient inclusion and exclusion criteria. For example, in contrast to the CAT, DEFINITE and DANISH trials, we did not exclude patients with valvular disease, those on anti-arrhythmics, or permanent atrial fibrillation patients, respectively (23, 25, 26). Increased mortality in our patient cohort may also be related to heart failure progression in our cohort or the type and severity of comorbidities (27). Cardiovascular disease, for example, is responsible for 30% more hospitalizations in outer regional and rural centers than urban centers. Age did not appear to be a factor (Table 2), which was different from many other published studies (15, 17, 18, 20). The possible role of regional, rural and urban health inequities to higher mortality requires further investigation.

### Only 19% of Patients Received Appropriate ICD Therapy

Of the 82 patients implanted with an ICD only 15 received appropriate pacing/shock therapy. On face value 18% appears to be a low success rate. However, in our study 67 patients did not experience ventricular arrhythmias over the duration of 4.8 years, which needs to be taken into consideration when determining a therapy's success rate. An appropriate therapy rate of 18% sits at the lower end of the published range of 11.6 to 24% over 2.1–3.64 years follow-up (11, 15, 19, 20, 28–31). In a large randomized control trial of 2,521 patients Bardy showed a 21% appropriate shock rate in the ICD arm (32, 33). However, many of these studies don't report the percentage of patients *who did not have sustained ventricular arrhythmias*. A unique and distinguishing feature of our study was its analysis of intracardiac electrograms allowing such determinations.

### Inappropriate ICD Therapy Was Associated With Poor Outcomes

We also found in our study that eleven patients (14%) received inappropriate therapy. This subset included 10 patients who had not experienced ventricular arrhythmias and one patient who had an arrhythmia. From our electrocardiogram analysis, inappropriate device therapy was also delivered in response to supraventricular tachycardias (Table 5). The inability of the device to distinguish between ventricular and atrial arrhythmias appears to be related to ICD programming and algorithms of rate parameters and detection times (34). This is an important area of future ICD development for primary prevention of sudden cardiac death because, in our study, those patients who received two or more inappropriate shocks were 18 times more likely to die than those who received only one event or no inappropriate therapy (Table 6). In a larger study, Van Rees similarly showed that inappropriate shock therapy was related to a 1.6-fold increase risk of mortality (35).

### NICM Patients had Significantly Higher Inappropriate Shocks

Another standout finding from the above analysis was the higher rate of inappropriate shock therapy delivered to NICM patients (20%) compared to ICM patients (4.9%; *p* = 0.039). This suggests that our NICM patients were predisposed to higher incidences of supraventricular or atrial arrhythmias. This group also had significantly higher incidence of obstructive sleep apnea (OSA) (23.7%) than ICM patients (0%) (*p* = 0.001). This is interesting because sleep apnea is strongly associated with atrial fibrillation (36), and was a predictor of inappropriate shocks in the study of Fernandez-Cisnal (30). Kreuz et al. also reported that ICD patients with sleep disorders had two times the inappropriate therapy of those without sleep apnea (37). Unfortunately, we did not examine the association between atrial fibrillation and sleep apnea throughout the 4.8-year follow-up. Why primary prevention NICM patients are more vulnerable to inappropriate shocks than ICM patients requires further investigation (26, 38). In addition, there is an urgent need to clarify heart failure guidelines for this vulnerability in NICM patients (4, 39).

### Risk/Benefit and Cost Considerations of ICD Implantation

Considering that 82% of total patients did not have sustained ventricular arrhythmias over the 4.8-year period, our study, although small, raises ethical questions about the pros and cons of having a device implanted, and validity of current ICD selection criteria. Notwithstanding, the life-saving potential of having a device, the clinical criteria for deciding who needs a primary prevention ICD is imprecise (8, 14, 40). Notwithstanding these important issues, three patients would have likely died without their ICD (Table 5). Healthcare costs are another consideration. The average procedural cost for ICD implantation in Australia is ~$22,000 (without complications), and $47,000 (with complications) (3, 41). In the Townsville district, the cost to healthcare providers for the 67 patients who did not need a device (no sustained ventricular arrhythmias) is estimated to be at least $1.5M, and if our data is representative of other centers in Australia, the national savings could be 82% of $155M per year (2011–2014) or $127M per annum (41).

### Limitations of the Study

A limitation of the present study was that it was a retrospective, observational analysis on a small population at a single center, regional tertiary hospital, that included both new implants and generator changes. A larger population of primary prevention ICD patients including more hospitals and service areas in Northern Australia, and a comparator from an urban center, would provide more information on the different rates of outcomes and to better evaluate this regional area compared to larger hospitals in metropolitan centers. Another limitation was that there was no analysis of ECGs performed in the patients who received appropriate shocks prior to implant which may have contributed to a more detailed assessment of a patient's risk. This is a topic of future research. Lastly, a detailed cost-effective analysis would provide important data to inform providers to the benefit and health-related quality of life of ICD recipients.

### Conclusions

Primary prevention ICD patients implanted at the Townsville University Hospital had high rates of mortality and low rates of sustained ventricular arrhythmias. The incidence of inappropriate ICD therapy was comparable to appropriate therapy, and was associated with increased mortality.

## Data Availability Statement

The raw data supporting the conclusions of this article will be made available by the authors, without undue reservation.

## Ethics Statement

The studies involving human participants were reviewed and approved by Townsville Hospital and Health Service Human Research Ethics Committee. Written informed consent for participation was not required for this study in accordance with the national legislation and the institutional requirements.

## Author Contributions

NE, GD, KN, and HL contributed equally to literature search, study design, data interpretation, and writing of the manuscript. All authors contributed to the article and approved the submitted version.

## Conflict of Interest

The authors declare that the research was conducted in the absence of any commercial or financial relationships that could be construed as a potential conflict of interest.
